# Extra Limites

**Published:** 1822-12-01

**Authors:** 


### EXTRA LIMITES.

### I.

### Dr. WILSON ON TIC DOULOUREUX.

*To the Editor of the Medico-Chirurgical Review.*

Sir,

From the attention which my letter on the subject of Morbid Sympathy, published in 1818, received from your Journal of January 1819, I take the liberty of transmitting to you the following case, which has recently occurred. In pages 255 and 279 on the subject of atonic rheumatism, I have suggested an opinion that the severe unmanageable disease designated tic douloureux, has its origin in the digestive organs, as the seat of its primary cause; and have there also mentioned some instances of recovery, in consequence of the complaint having been treated on this principle. The accompanying case is so decisive on that point, that I am willing to lay it before you, seeing that if you choose to give it to the public in your Journal, it may, perhaps, have the effect of drawing the attention of others to the subject, and lead to a more effective practice in treating this cruel disease.

*Case.* Peter Storry, a servant, ætat. 58, is affected with tic douloureux in an excruciating degree. He describes the pain as always commencing at a point in his upper lip on the right side, where it is joined by the ala of his nose; from whence it spreads upward with great violence, shooting along his cheek to his temple, and over the whole side of his head, the pain being so severe as to make him cry out in great agony. It attacks him repeatedly in the course of the 24 hours, in paroxysms of several hours duration, and even during the intervals the pain remains with very considerable severity. He has been subject to occasional attacks of the same kind, in a less degree, for sixteen years, of shorter duration than the present, and with intervals of some weeks, or even many months, at a time; and he has observed that exposure to cold, and wet weather very readily excited a return.

The present fit of the disease came on about the middle of May last, without his being able to attribute it to any occasional cause; since which time to the present day,

(July 5th,) his time has passed under constant severe pain, and part of every day under intense torment—at present his pulse is natural, and his skin cool; his tongue is loaded with a thick membranous fur, in so far as it is visible; as the smallest motion of his tongue or lips in attempting to speak, or take in food, is certain of exciting a paroxysm, in consequence of which deprivation, together with want of sleep, his strength has become greatly impaired. He has been following medical advice at home for six weeks past, during which time he has used laxatives freely, chiefly saline, with some doses of calomel, he has also had an emetic or two, which did not operate freely: these have been followed with carbonate of iron in considerable quantity, viz. one drachm thrice a day for two weeks—of late this has been changed for the arsenic solution, taken freely without any benefit.

On the 5th of July he was admitted a patient in the Dispensary here; and came under my care. It appearing quite certain, on examination, that his digestive organs remained loaded with an accumulation of morbid contents, and believing that the primary cause of his misery still existed there, notwithstanding the extensive evacuations which had been procured, by the lenient purgatives already administered, it became necessary to have recourse to some more powerful remedy—accordingly, with the concurrence of the other medical gentlemen, he was directed to take the following bolus next morning—calomel gr. vij. antimon. tartar. gr. i. ℳ. ft. p. duo. By this dose he vomited a considerable quantity of dark coloured corrupted bile, and had three very offensive stools.

He slept a little in the succeeding night, and passed the next day, with some mitigation of his complaint, in thus far—that although the lancinating pain shooting from his lip along his cheek and temple, were as frequent as ever; yet they were less severe, and he had no regular excruciating paroxysm.

The bolus to be repeated every other morning—also to receive an enema, with fifty drops of tinct. opii, every night.

By the second and third doses, he vomited dark coloured bile, each day, and voided offensive stools, mixed with a great quantity of hard scybalæ.

By the fourth and fifth doses he continued to vomit unhealthy looking bile, mixed with viscid phlegm—he also passed offensive stools mixed with scybalæ as before. He is now much relieved, being able to speak, and to swallow food without exciting a fit of pain, and rests better in the night; his tongue is considerably cleaned from the thick fur, and he is able to walk about in his room; there are some slight returns of pain along the side of his face, but no regular paroxysm for eight days past. By the sixth and seventh doses, the discharges were less offensive, assuming a more healthy appearance, his tongue is clean, and his night's rest natural; the occasional transient pains slight, and less frequent, is still gaining strength, being now able to walk out.

July 29th. Ordered to omit the mercurial bolus; continuing the sedative enema; and to take pulv. cinchon. ℥j. every four hours, in small doses, with an opening pill occasionally if necessary.

Aug. 10. Continues better—is permitted to return home—continue the p. cinchona, with one grain of opium, every night, in place of the sedative enema.

Sept. 1st. Has used his medicines regularly, is much stronger, being now able to undertake easy work—ordered to omit the cinchona, and use the following tonic electuary: limat. ferri ℥i. crem. tart. ʒiij. pulv. cinchon. ℥iss. pulv. zinziber. ʒiss. syrup. com. ℥ii. ℞. ft. elect. A small tea-spoonful three times a day.

Sept. 15. Continues free from pain, and has acquired a very healthy appearance—repeat his medicines.

Oct. 2d. Continues well—dismissed.

It may be remarked, on the above case, that as it is decidedly one of morbid sympathy, it is likely enough the cure may not be permanent, but that it may afterwards recur in this man, if a similar primary cause shall take place in the primæ viæ; but should this happen, the nature of the disease being known, the remedy is at hand. That tic douloureux is in every instance a disease of morbid sympathy cannot with certainty be said: but, from having observed the very extensive power of that law in the system, and judging from what I have witnessed of this disease, as well as from what I can gather from the recital of others, I esteem it a high probability, indeed next to a certainty, that it always is so.

I am, Sir, respectfully yours,

ANDREW WILSON, M.D.

*Senior Phys. to Kelso Dispensary.*

*Kelso, Oct. 15th,* 1822.

II.

### *Case of the late Mr. Knox, Surgeon, of Great Russel Street, with the Appearances on Dissection. By J. Johnson, M.D.*

It may seem a paradox, but I think the following dissection will prove it to be an incontestible fact, that the magnitude of an organic disease will sometimes render it incognizable by the physician and anatomist, during the life of the patient.

The late Mr. Knox, Surgeon, in Great Russel Street, had been for several years in an infirm state of health, owing, as he imagined, to a liver complaint. The prominent features of his disorder were, indigestion, sickness at stomach, and an irregular state of the bowels, attended with loss of flesh and strength. Latterly, and when obliged to confine himself to the house, gastric irritability, uneasiness in the region of the stomach, and a dysenteric affection of the bowels were the predominant sources of complaint. At this time, viz. in January and the beginning of February 1820, Mr. Knox was visited by several of the most eminent physicians, and also anatomists of this metropolis—and they unanimously declared that there were no evidences whatever of any enlargement or organic disease of the *liver*. I did not see the patient till the day before his death, and the surface of the abdomen being raw from the effects of a blister, I made no manual examination, and hazarded no opinion on the nature of the case. To the eye there was no appearance of an enlarged liver. He died the next day, and I had permission to examine him. The following minutes I sent to some of the medical gentlemen who had seen Mr. Knox before his death, but who were not present at the examination.

" *Minutes of Dissection.—Present, Dr. Armstrong, Mr. Ogle, Mr. Plumbe, Mr. Dickenson, and Dr. Johnson. 14th Feb.* 1820. Considerable emaciation—sallowness and slight yellowness of the skin. On laying open the abdominal cavity, a quantity of yellowish serum ran out. The liver was enormously enlarged, filling both hypochondria, the epigastric region, and stretching from the ribs of one side to the ribs of the other. It also extended downwards to near the umbilicus. It was studded, both on the convex and concave surface with irregular-sized white tubercles, not very prominent, except at one part of the concave surface. The substance of the liver being cut into, presented a structure almost totally morbid. The tubercular masses were in various stages : some of them pretty firm, white, and homogeneous; others soft, and almost gelatinous, while considerable masses of them were broken down into a fluid resembling pus or cream. From one of these depôts a continued stream of this purulent-looking fluid issued, on being cut into by the knife.

There were very few traces of the original parenchymatous structure of this organ remaining, and these portions were pale, flabby, and thinly distributed among the disorganized portions. There was some thin watery bile in the gall-bladder. On the under surface of the diaphragm, and exactly opposite to a rather prominent and irregular tuberculated mass, was an elevated morbid structure, very hard, irregular, and precisely corresponding in size, with the projecting tubercular mass on the liver. It appeared to be caused by the irritation of the latter.

The stomach appeared sound externally, but the villous coat was as smooth and pale as a sheet of writing paper. It could be easily peeled off from the subjacent muscular coat. The pyloric orifice was little, if at all, contracted; but its parietes were enlarged and indurated into a solid and irregular scirrhous mass. It is curious that exactly opposite the pylorus, and in contact with it, was a ruggid, hard, and irregular tubercular projection of the liver, and which may be considered as having long kept up a degree of irritation upon that part of the stomach which was in contact with it. This idea is strengthened by what was seen on the diaphragm, as above stated. There was no ulceration of the pylorus. The pancreas was somewhat enlarged, and appeared to partake a little of the morbid structure of the liver.

The first order of mesenteric glands were enlarged and intensely indurated, appearing like a thick-set row of white beads strung all along the interior curvatures of the small intestines. The other mesenteric glands were not much affected. The large intestines were irregularly contracted and dilated in several places. There were ulcerations in several parts of the mucous membrane of the large intestines. The thoracic viscera were sound."

It is almost needless to remark that the uniform expansion of the biliary organ over the epigastric and hypochondriac regions, its thin edge losing itself among the intestines in the umbilical region, was the cause why no enlargement of the liver could be detected by the hands of physicians, surgeons, and teachers of anatomy, during the life of the patient. This statement, therefore, is not meant to insinuate any want of discrimination on the part of the medical attendants, but to show what sources of fallacy we have to contend with, in the investigation of diseases, and how cautious we should be of giving hasty and decided opinions on the nature of internal lesions. It is remarkable, but it is reasonable enough, that the *confidence* of our decisions, on these occasions, is generally in an inverse ratio to our years and experience. The perusal of the above case may teach the juniors of the profession *humility*, which is an attribute of wisdom, and induce them to be not only cautious themselves, but merciful and charitable to their brethren.

I think an attentive consideration of the above appearances would lead us to trace the origin of the disease to a tuberculated state of the liver, and to view the other lesions, functional and organic, as consequences of this state. It may, however, admit of doubt whether or not the induration of the mesenteric glands was coeval with the disorganization in the liver. The disease was evidently incurable from the beginning; and it is sufficiently obvious that any very active mercurial treatment would be more likely to do harm than good. I may be permitted to make one more remark. The size of the abdomen, during life, could not have been in proportion to the great emaciation of the body generally:—and although this, of itself, would not lead us to suspect enlargement of the liver, it was a strong indication of disease of structure in some part or parts of the abdomen.

JAMES JOHNSON.

*Spring Gardens*, 1822.

---

III.

Sir, *Lincoln, Sept.* 13*th*, 1822.

I have taken the liberty of sending you the following case, as it appears to me to confirm the statement of M. Richerand which is noticed in the 427th page of your excellent Review. J. SWAN.

Joseph Ashton, ætat. 48, was admitted into the County Hospital in October 1821, with a cancerous disease, which occupied the right angle of the mouth, and extended over the whole of the lower lip. It was evident the disease must soon prove fatal; and it was the desire of the patient that I should try to relieve him. I began my incision at the upper lip, as near to the angle of the mouth as the disease would allow; and continued it very near to the masseter muscle. I then made another incision obliquely towards the chin, and removed the whole of the lower lip, nearly as low as the chin. I separated the small remaining part from which I removed the lip with the scalpel, from the jaw, to allow of its being drawn towards the upper lip. I brought the parts together with sutures, but nearly the whole wound flew open; nevertheless it healed very well, and the part supplying the place of the lower lip was drawn considerably towards the upper one, so that the mouth performed all its functions well, and had a much better appearance than could possibly have been expected. Had the removal of the lower lip occasioned much inconvenience, it was my intention to have made a new one from a portion of the skin under the chin.

